# Distribution of nuchal translucency thickness at 11 to 14 weeks of gestation in a normal Turkish population

**DOI:** 10.3906/sag-2001-48

**Published:** 2021-02-26

**Authors:** Gülseren DİNÇ, İlker EYÜBOĞLU

**Affiliations:** 1 Department of Obstetrics and Gynecology, Faculty of Medicine, Karadeniz Technical University, Trabzon Turkey; 2 Department of Radiology, Faculty of Medicine, Karadeniz Technical University, Trabzon Turkey

**Keywords:** uchal translucency, ultrasonography, first trimester, crown-rump length, ethnic origin

## Abstract

**Background/aim:**

The aim of this study was to determine fetal nuchal translucency (NT) thickness nomogram values in first trimester in a Turkish population and compare them with previously reported European and Asian nomogram data.

**Material and methods:**

Ultrasonographic measurements of crown-rump length (CRL) and NT thicknesses were obtained from 11 to 14 weeks of gestation in a normal Turkish population. Pregnant women with singleton pregnancy and fetal CRL between 45 and 84 mm were included in the study. The mean 1st, 3rd, 5th, 50th, 90th, 95th, 97th, and 99th percentiles and fixed cut off values of ≥ 2.5 mm, ≥ 3 mm, ≥ 3.5 mm NT thicknesses for a CRL between 45 and 84 mm were determined.

**Results:**

A total of 1605 healthy fetuses were enrolled in the study. The sonographic measurements were performed on 1541 (%94) fetuses transabdominally and on 99 cases (%4) by the transvaginal route. The mean NT thickness for CRL between 45 and 84 mm was 1.57 ± 074 mm, and the mean 95th, 97th, and 99th percentiles of these values were 2.82 mm, 3.17, and 4.75 mm, respectively. The incidence of NT thicknesses at fixed points of ≥ 2.5 mm, ≥ 3 mm, and ≥ 3.5 mm in normal fetuses were 6.7%, 4.1%, and 2.1%, respectively.

**Conclusion:**

The present study demonstrated the nomogram data of fetal NT thickness in a Turkish population. We think that this report will be useful for further research related to NT thickness values on the prenatal diagnosis for the first trimester chromosomal abnormalities in Turkish populations.

## 1. Introduction

Nuchal translucency (NT) is the strongest and the most commonly used sonographic parameter in first-trimester screening for chromosomal abnormalities especially for Down syndrome and fetal structural abnormalities including major cardiac malformations, certain genetic syndromes, and high risk of miscarriage and intrauterine fetal death [1–3]. All these risks increase in proportion to the increase in nuchal translucency thickness [1,4].Combining NT thickness with maternal age and serum concentrations of free beta-human chorionic gonadotropin and pregnancy-associated plasma protein-A, it is possible to detect at a rate up to 90% of Down syndrome cases with a false-positive rate of 5% [5].

Some studies demonstrated that there was a significant difference in the first trimester of nuchal translucency measurements between fetuses of different ethnic origins [6,7]. Therefore, it may not be appropriate to use published NT values or a certain cut-off value for the Turkish population to evaluate chromosomal and structural abnormalities. In our literature review, we found only one study with low number of cases about NT nomogram data in a Turkish population [8].

The aim of this study was to determine fetal NT thickness nomogram values for euploid fetuses at 11–14 weeks of pregnancy in a Turkish population and to compare them with the literature data.

## 2. Materials and methods

This study was conducted retrospectively at Karadeniz Technical University Farabi Hospital, a tertiary hospital in our region. Pregnant women with 11–14 weeks of gestation who referred for the first trimester screen between January 2010 and October 2019 were included in the study. The study protocol was approved by the Karadeniz Technical University Faculty of Medicine Ethical Committee and the institutional review board responsible for all patient data and images available in hospital information system. Additionally, informed consent was obtained from all the patients included in the study. Examinations were performed with the Voluson 730 expert (General electric, Waukesha, Wisconsin) ultrasonography device. The CRL and NT measurements were mainly performed by transabdominal route. In fetuses poorly visualized transabdominally, the measurements were performed with transvaginal examination. First trimester screen and measurements of NT and CRL were done by certified specialist physicians with at least 3-year experience in obstetric ultrasonography.

US measurements of CRL and NT were performed between 11 and 14 weeks of gestation in a Turkish population. Inclusion criterion for nomogram study was pregnant women with singleton pregnancy between gestational ages 11 to 14 weeks with CRL between 45 and 84 mm. Cases with congenital anomaly, karyotype abnormalities, multiple pregnancies, and fetuses with abnormal US scan were excluded from the nomogram study. NT and CRL measurements were performed according to the guidelines outlined by the Fetal Medicine Foundation [9,10]. Ultrasonographic examinations were performed by skilled physicians for first trimester screen. CRL measurement carried out in a mid-sagittal view ideally in which the whole embryo or fetus oriented horizontally on the screen. NT measurements were performed on magnified mid-sagittal images so that head and upper thorax of the fetus only occupy three-quarter of the screen. This is defined by the view of echogenic tip of nose, rectangular shape of zygomatic and maxillary bones, translucent appearance of diencephalon in the center and the NT in posterior.

### 2.1. Statistical analysis

Statistical analysis was performed using SPSS version 23. The fetuses with CRL between 45 and 84 mm were analyzed. NT thickness based on 5 mm and 10 mm intervals of CRL of the fetuses, and the mean, standard deviation for each intervals and reference values for each 1st, 3rd, 5th, 50th, 90th, 95th, 97th, and 99th percentiles of the NT thickness were calculated. Pearson’s correlation coefficient was used to measure the association between NT and CRL. Statistical significance was defined as P < 0.05.

## 3. Results

First trimester screen was done in 1640 singleton pregnancies. The measurements were performed on 1541 (%94) fetuses by the transabdominal route and on 99 cases (%4) by the transvaginal route. Thirty-five (%2.1) cases with unsatisfactory evaluation according to the Fetal Medicine Foundation guideline were excluded. A total of 1605 fetuses were used in the final analysis. Then maternal age was between 18 and 47 (mean 30.2 ± 5.4) years. When the values above the 97th percentile for NT thickness were accepted as pathological values, pathological values for 11–11^+6^, 12–12^+6^, and 13–13^+6^ weeks of gestation were calculated as 3.10 mm, 3.20 mm, and 3.30 mm, respectively (Table 1). At the gestational age of 11–14 weeks, the reference values for the 99th percentile were calculated as 4.75 mm. The percentile values of the NT thicknesses for 5 mm and 10 mm CRL intervals were separately calculated (Tables 1 and 2). 

**Table 1:** The percentile values of the NT thicknesses for 10 mm CRL intervals.

Percentiles												
Weeks	CRL	n	mean ± SD	1%	3%	5%	10%	50%	90%	95%	97%	99%
11–11+6	45–54	355	1.40 ± 0.79	0.60	0.70	0.79	0.80	1.28	2.00	2.60	3.10	5.89
12–12+6	55–68	820	1.58 ± 0.76	0.80	1.00	1.00	1.00	1.40	2.00	2.60	3.20	5.43
13–13+6	69–84	430	1.68 ± 0.64	0.90	1.00	1.00	1.10	1.70	2.44	2.70	3.30	4.57
Total (11–13+6)	45–84	1605	1.57 ± 0.74	0.70	0.80	0.90	1.00	1.60	2.10	2.82	3.17	4.75

CRL = Crown-rump length, NT = Nuchal translucency

**Table 2:** The percentile values of the NT thicknesses for 5 mm CRL intervals.

Percentiles											
CRL	n	mean ± SD	1%	3%	5%	10%	50%	90%	95%	97%	99%
45–49	115	1.25 ± 0.49	0.60	0.62	0.70	0.80	1.00	1.80	2.12	2.77	3.46
50–54	240	1.47 ± 0.88	0.60	0.70	0.80	0.80	1.30	2.00	2.70	3.92	6.53
55–59	319	1.58 ± 0.82	0.80	0.93	1.00	1.00	1.40	2.00	2.30	3.00	7.20
60–64	279	1.56 ± 0.73	0.88	0.94	1.00	1.00	1.40	2.00	2.70	3.20	5.24
65–69	257	1.59 ± 0.67	0.85	1.00	1.00	1.10	1.40	2.30	2.60	3.22	4.67
70–74	195	1.62 ± 0.56	0.80	0.90	1.00	1.10	1.50	2.40	2.50	2.63	4.33
75–79	134	1.80 ± 0.71	0.90	1.00	1.00	1.15	1.70	2.70	3.22	3.99	4.92
80–84	66	1.61 ± 0.83	0.90	0.90	1.00	1.10	2.00	2.30	4.32	4.70	4.70
Total (45–84)	1605	1.57 ± 0.74	0.70	0.80	0.90	1.00	1.60	2.10	2.82	3.17	4.75

CRL = Crown-rump length, NT = Nuchal translucency

In the present study, mean NT measurements at 11–11^+6^, 12-12^+6^, and 13–13^+6^ gestational weeks were 1.40 ± 79 mm, 1.58 ± 0.76 mm, and 1.68 ± 0.64 mm, respectively. NT thickness for the 95th, 97th, and 99th percentiles were 2.82 mm, 3.17 mm, and 4.75 mm, respectively. The Pearson correlation analysis showed a significant and positive correlation between NT and CRL (r = 0.131 and P < 0.001).

Table 3 shows the distribution of the NT thickness with gestational age according to 2.5 mm, 3 mm, and 3.5 mm fixed cut off values. In 11–11^+6^ weeks of gestation, 5.3% of study population has 2.5 mm or greater NT thickness, and it increased up to 10% in 13–13^+6^ weeks of gestation. When we take 3 mm and 3.5 mm as fixed cut off values for NT thickness, 4.1% and 2.1% of population were above the threshold.

**Table 3:** The distribution of nuchal translucency measurements according to 2.5 mm, 3 mm, and 3.5 mm cut-off values.

			NT ≥ 2.5mm		NT ≥ 3mm		NT ≥ 3.5mm	
Weeks	n	(%)	n	(%)	n	(%)	n	(%)
11–11+6	355	22.1	19	5.3	11	3.1	6	1.7
12–12+6	820	51.1	43	5.2	35	4.3	15	1.8
13–13+6	430	26.8	46	10.7	20	4.7	13	3.0
Total (11–13+6)	1605	100	108	6.7	66	4.1	34	2.1

NT= Nuchal translucency

## 4. Discussion

The present study demonstrated that fetal NT thickness increases with CRL measurements in the Turkish population, which is compatible with previous reports [6,8,11–16]. In our study, at CRL between 45 and 84 mm, the mean NT thickness was 1.57 mm. The NT thicknesses of 95th, 97th, and 99th percentiles were 2.82 mm, 3.17 mm, and 4.75 mm. In prenatal Down syndrome screening, three cut-offs 2.5 mm, 3 mm, and 3.5 mm have been proposed for NT thickness. [17,18]. When we take these three cut-offs as indicators of increased risk of chromosomal aberration, 6.7%, 4.1%, and 2.1% of our study population were above the threshold. We suggested that false-positive rate increases with increasing CRL and/or gestational age; therefore, each NT measurement should be obtained according to the gestational age for screening chromosomal abnormalities.

Several studies (Table 4) have reported nomogram values of NT thickness in the first trimester [6,8,11–16]. Thilaganathan et al. [6] reported first trimester fetal nuchal translucency measurements at 10–14 weeks in women from different ethnic groups. The mean NT thicknesses in Caucasian, African, Asian, and Caribbean populations were 1.54 mm, 1.48 mm, 1.61 mm, and 1.51 mm respectively [6]. The differences are small and the results are very close to ours, which is 1.57 mm. Thilaganathan et al. stated that although there are significant differences in nuchal translucency values between ethnic origins in Asian population, these differences are too small to require any correction [6].

**Table 4:** Results of previously reported studies estimating reference values for nuchal translucency in different ethnic origins.

Ethnicorigin	n	GW	CRL Range/mean (SD)	Mean NT mm (SD)	Median NT mm (SD)	NT 95th per.mm n (%)	NT97th per.mm	NT 99thper. mmn (%)	NT ≥ 2.5 mmn (%)	NT ≥3 mmn (%)	NT ≥ 3.5 mmn (%)
Caucasian6	1031	10–14	58.0 (10.3)	1.54 (0.15)	1.02 (0.43)	-	-	-	-	-	-
African6	449	10–14	60.0 (10.7)	1.48 (0.49)	0.97 (0.30)	-	-	-	-	-	-
Asian6	232	10–14	61.0 (10.0)	1.61 (0.86)	1.05 (0.55)	-	-	-	-	-	-
Caribbean6	232	10–14	58.9 (9.7)	1.50 (0.92)	0.99 (0.57)	-	-	-	-	-	-
Thai11	4352	10–14	60.2 (9.7)	1.15 (0.38)	1.15-1.85	1.57–2.10	-	-	-	-	-
Asian12	879	9–14	53.1 (15.2)	1.72 (0.49)	1.7 (0.5-4.3)	2.32–2.83	-	-	53 (6)	-	-
Korean13	2577	11–14	60.1 (9.07)	1.62 (0.50)	1.6	2.24–3.03	-	-	103 (4)	-	-
Japanese14	970	11–14	45-80	1.2-1.9	-	2.10–3.2	-	-	-	-	-
Danish15	222 505	11–14	45-84	-	-	1.6	2.8	4.0	-	-	-
London16	20 217	11–14	38-84	-	-	2.32–2.83 917 (5)	-	221 (1)	106 (5)	-	-
Turkishprev. rep.8	190	11–14	63.63 (10.0)	1.23 (0.43)							
Turkishpresent study	1605	11–14	45–84	1.57 (0.74)		2.82	3.17	4.75	108 (6.6)	66 (4)	36 (2.2)

GW= Gestational week, CRL = Crown-rump length, NT = Nuchal translucency, prev. rep. = Previous report, per. = Percentile.

Kor-Anantakul et al. [11] reported that the mean NT thickness was 1.15 mm in the first trimester fetuses in Thailand population. In the same study, at a CRL below 85 mm, at the 95th percentile, the NT thickness of 2.10 mm was thinner than the accepted fixed cut-off point of ± 2.5 mm [11]. They concluded that the NT thicknesses in healthy Thailand fetuses were found to be thinner than those of the other Asian populations [11]. In our study, the 95th percentile NT thickness in the first trimester was between 2.12 and 4.32 mm. The mean NT thickness value of 2.82 mm was higher than the accepted fixed cut-off point of ≥ 2.5 mm. Additionally, 6.7%, 4.1%, and 2.1% of the first trimester fetuses had higher NT thicknesses when we accepted fixed cut-off points above 2.5, 3, and 3.5 mm.

In another Asian study, the incidence of NT ≥ 2.5 mm ranged from 0% with CRL < 30 mm up to 21.6% in the subgroup with CRL ≥ 80 mm [12]. In the present study, the mean incidence of NT ≥ 2.5 at CRL between 44 and 85 mm was 6.7%. Chung et al. [13] demonstrated that the 95th percentiles of the NT thicknesses for a CRL between 45 and 80 mm ranged 2.24 to 2.93 mm, and the mean NT thickness was 1.62 mm in a healthy Korean fetus. In the same study, the incidence of NT thickness greater than or equal to 2.5 mm ranged from 2.2% to 12.5 % in the first trimester fetuses [13]. Four percent of the fetuses had NT thicknesses greater than or equal to 2.5 mm comparable with our value of 6% [13]. Hasegawa et al. [14] reported the nomogram values in a Japanese population with the 95th percentile ranging 2.1 to 3.2 mm. These values are slightly lower than our results ranging from 2.12 to 4.32 mm.

In European-based studies, NT nomogram results are close to those of Asian countries [15,16]. In Pandya et al.’s multicenter study [16] composed of 20,217 first-trimester fetuses, 5% and 1% of the fetuses had NT thicknesses above 95th and 99th percentiles, and at a 2.5 mm fixed cut off point, 106 (5%) of the healthy fetuses were above this cut-off value. In the same study, nuchal translucency values were above the 95th percentile in 77% of fetuses with trisomy 21 and 78% with other chromosomal abnormalities [16]. In a Danish study, 222,505 euploid fetuses from 17 national departments underwent routine first-trimester screening, and the fetuses were divided into 3 groups according to prenatal NT measurements: NT < 95th (97.6%) percentile, NT 95th–99th (2.1%), and NT > 99th percentile (0.3%). The mean NT measurements were 1.6 mm, 2.8 mm, and 4.0 mm in the first, second, and third groups, respectively [15].

When compared with the literature data, our average NT thickness values were found to be slightly higher than those of Japanese and Thai populations and close to those of other Asian countries (Table 4). NT thickness measurements and percentiles obtained from multicenter Danish and London-based studies from Europe were found to be consistent with our study data [15,16]. Several other studies demonstrated that these ethnic differences are too small to require correction [6]. In other words, the ethnic differences in NT thickness measurement were not clinically significant when the NT is used in screening for Down syndrome although there are some differences in NT values among the ethnic populations [6,14–16].

There were some limitations of the present study. The first and main limitation is the retrospective nature of the study. The second limitation is that data were obtained from a single university and included only fetuses in a particular region of the country (Eastern Black Sea Region). The third limitation is the absence of long-term follow-up results of normal-born fetuses with increased NT thickness values above 95th, 99th percentiles or above of fixed cut-off values of 2.5 mm, 3 mm, and 3.5 mm. Senat et al. [18] reported the results of long-term outcome of fetuses born whose first-trimester NT measurements were at the 99th percentile or above. They confirm that fetuses with increased NT thickness above the 99th percentile and normal karyotype have high risk of adverse perinatal outcome. However, no negative prognosis was detected in 2-year follow-up of living children [18].

In conclusion, the present study demonstrated the nomogram data of fetal NT thickness in a Turkish population. This is the largest study on NT nomogram data in first-trimester normal fetuses in a Turkish population. We believe this report will be useful for further research related to NT thickness values on the prenatal diagnosis for the first-trimester chromosomal abnormalities in Turkish populations. 
